# Investigation of the temporal roaming behaviour of free-roaming domestic dogs in Indigenous communities in northern Australia to inform rabies incursion preparedness

**DOI:** 10.1038/s41598-019-51447-8

**Published:** 2019-10-17

**Authors:** Elizabeth K. Maher, Michael P. Ward, Victoria J. Brookes

**Affiliations:** 1 Sydney School of Veterinary Science, The University of Sydney, Camden, Australia; 2 School of Animal and Veterinary Sciences, Charles Sturt University, Wagga Wagga, Australia

**Keywords:** Diseases, Risk factors

## Abstract

Australia is canine rabies free but free-roaming, domestic dog populations in remote northern communities are at risk of an incursion due to proximity to rabies-endemic south-east Asia. Unrestricted contact between dogs could facilitate rabies spread following an incursion, and increase the impact on both dogs and people. Whilst dog vaccination is the foundation of rabies prevention, control strategies could be enhanced by understanding the temporal pattern of roaming and associated risk factors, so that movement restrictions can be targeted. Global positioning system datasets from 132 dogs in eight Indigenous communities in the Torres Strait and Northern Peninsula Area (NPA) of Australia were analysed using regression methods. The influence of risk factors (including age, sex, location, season and hour of day) on dogs’ distance from their residences were assessed. Dogs roamed furthest in the NPA and during the dry season. Daily peaks in mean roaming distance were observed at 1000–1100 hrs and 1700–1800 hrs in the Torres Strait, and 1700–1800 hrs in the NPA. These findings demonstrate that understanding community-specific temporal roaming patterns can inform targeted movement restrictions during an outbreak of rabies in remote communities in northern Australia.

## Introduction

Rabies is distributed throughout Asia, Africa, Europe and North and South America^1^. Although rabies virus can infect all mammals, canine-rabies is the most widely distributed form and accounts for over 95% of human infections^2,3^. It causes an estimated 59,000 human deaths, and costs USD 8.6 billion annually due to premature deaths, post-exposure prophylaxis (PEP) and income loss during courses of PEP^4^. Improvements in control and prevention – such as bite prevention, vaccination, surveillance, public awareness and effective dog movement restrictions – have the potential to significantly reduce the impact of rabies^4^.

Australia is currently free of canine-rabies^3^ but the proximity of northern Australia to rabies-endemic Indonesia places this area at risk of an incursion^5^. Outbreaks in south-east Asia have demonstrated the propensity for transboundary spread^6,7^, and recent research has identified potential entry and transmission routes for canine-rabies into Australia via Indonesia, Papua New Guinea and the Torres Strait^5,8,9^.

Domestic dogs are abundant in many Aboriginal and Torres Strait Islander (Indigenous) communities in northern Australia^10,11^. Although owned, these dogs roam freely, posing a risk in the case of a rabies incursion due to the potential for high contact rates^10^. Overlap between wild-dog territory and roaming ranges of domestic dogs also presents a risk of canine-rabies transmission between these populations^12^, and increases the risk of endemicity should an incursion occur^5,13^.

Potential spread and the impacts of rabies in Australia have been investigated using rabies-spread models in populations of both free-roaming domestic and wild-living dogs^14–17^. Whilst the size and duration of predicted outbreaks vary between models, as well as between locations and types of dog population, predictions have consistently been sensitive to contact rates. Rabies is a vaccine preventable disease and mass vaccination of dogs is considered the foundation for canine-rabies control and elimination^18^; it has been demonstrated to reduce incidence in many regions^19^. However, due to the predicted influence of contact rates on disease spread, adjunct measures such as movement control could also reduce the potential for spread.

Accordingly, the Australian Veterinary Emergency Plan for response should a rabies incursion occur recommends a range of control measures^20^. As well as vaccination, strategies include quarantine of suspected infected and exposed dogs and movement restrictions of all dogs. Research has shown that whilst movement restrictions would generally be supported by northern Australian communities^15^, effective implementation would be difficult due to material disadvantage in this region^21^. Therefore, given limited resource availability, targeted movement controls – for example, movement restriction of types of dogs that are more likely to roam, or restriction of all dogs at times when roaming is common – could be a more effective strategy than blanket movement restrictions. Understanding the temporal roaming patterns of free-roaming domestic dogs in northern Australian communities is needed to develop such strategies.

Previous research on free-roaming dogs in northern Australia^12,22–25^ and elsewhere has focussed on spatial patterns. For example, Hudson *et al*.^23^ demonstrated that free-roaming dogs could be categorized by the size of their home range and the duration required to reach maximum home range size, Oesch^26^ demonstrated variation in the home range size and speed of roaming, and Kennedy *et al*.^27^ observed increased activity by dogs at dawn and dusk. Research on temporal roaming patterns of free-roaming dogs is very limited.

The objective of this study was to describe the temporal roaming patterns of free-roaming domestic dogs in northern Australian Indigenous communities in terms of their distance from their residence, and investigate determinants that could influence the distance, duration and temporal patterns of roaming. Our overall aim was to generate information to guide strategies to restrict dog movement should an incursion of rabies occur in northern Australia. It was hypothesised that the temporal roaming patterns of free-roaming domestic dogs in remote Indigenous communities is not random, and is influenced by determinants associated with the communities, individual dogs, season and the time of day.

## Methods

### Dataset

**Figure 1:**
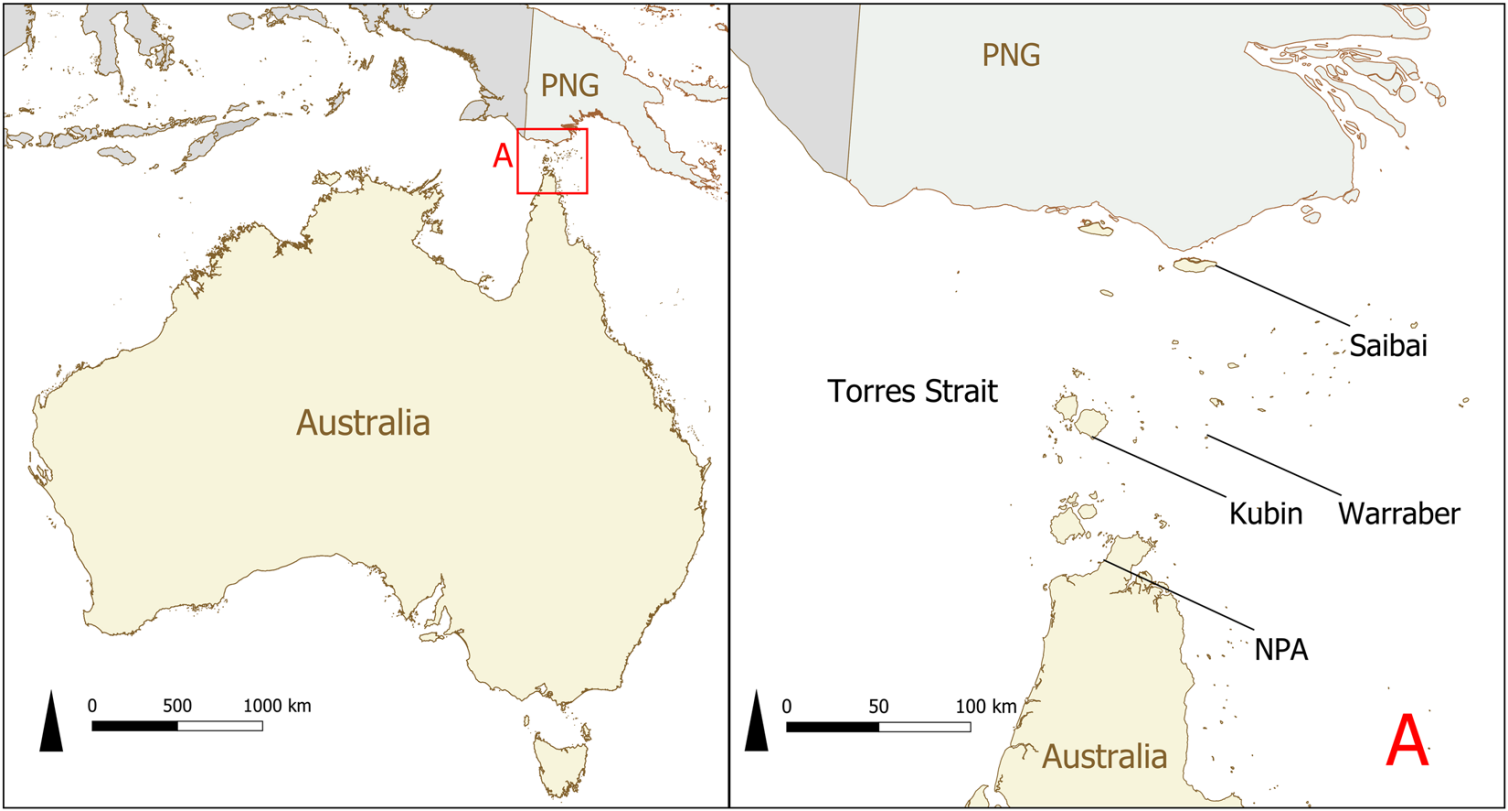
Map of the location of the study regions (**A**) in a study of the temporal roaming patterns of free-roaming domestic dogs in the Torres Strait and Northern Peninsula Area, Queensland, Australia. NPA = Northern Peninsula Area, PNG = Papua New Guinea.

Data were collated from existing GPS telemetry datasets collected from free-roaming domestic dogs in Indigenous communities in the Torres Strait (TS) and the Northern Peninsula Area (NPA) of Queensland, northern Australia (Fig. 1) between September 2013 and March 2017 (Table 1). The NPA comprises five communities with a total estimated population size of 800 dogs^11^. The Torres Strait lies between Papua New Guinea and mainland northern Queensland and comprises many islands of which 17 are inhabited. The Torres Strait communities included in the current study were Warraber, Saibai and Kubin, with estimated population sizes of 41, 43 and 39 dogs, respectively^28^.

**Table 1:** Study sites, dataset collection months and human demographics in a study of temporal activity of free-roaming domestic dogs in the Torres Strait and Northern Peninsula Area (NPA), Queensland, Australia.

Region	Community	Human Population	Proportion Indigenous	Study months
Northern Peninsula Area	Bamaga^48^	1,164	80.2% (NPA region)	September 2013, April 2014 and September 2014 (NPA region)
	Injinoo^49^	561		
	New Mapoon^50^	383		
	Seisia^51^	260		
	Umagico^52^	427		
Torres Strait	Kubin^53^	187	100%	December 2016
	Saibai^54^	456	85.6%	March 2017
	Warraber^55^	245	96.7%	September 2016

Domestic dogs in the study communities are owned and free-roaming^11,28^. Very few, if any, dogs are stray (unowned) and dogs do not generally change ownership. Owners feed commercial dog food and scraps to their dogs, and dogs also scavenge food whilst roaming (from bins, school lunches, the council refuse dump and from other residents). Environmental health workers provide limited healthcare such as parasiticides for dogs, and veterinary healthcare (for example, surgical or chemical sterilisation) is provided intermittently^21,29^.

Details of the methods used for data collection are included in Bombara *et al*.^12^, Durr and Ward^24^, Durr *et al*.^25^, and Brookes *et al*.^28^. The study and methods were implemented in accordance with, and were approved by, the Human Ethics Committee (#2013/757) and the Animal Ethics Committee (#N00/7–2013/2/6015) of The University of Sydney.

Briefly, datasets were collected using GPS recording units (CatTraQ™, http://www.mr-lee.com/, accessed 1.12.18) that were attached to nylon collars and fitted to free-roaming domestic dogs at study sites. Dog selection was guided by local Environmental Health and Animal Management Workers. In the NPA, 159 dogs were collared in 5 communities during three collection periods. Mean duration of datasets was 56.2 hours (sd 11.3 hours). In the TS, 76 dogs were collared in three communities during three collection periods. Mean duration of datasets was 69.5 hours (sd 37.1 hours). GPS ‘fix interval’ was 1 minute in the NPA and 15 s in the TS; these differences were a result of the different objectives of the original studies. ‘Fix intervals’ are the specified duration after the previous location recording (‘fix’) at which the unit starts to search for satellites to fix the next location.

### Data management

A description of individual dogs was collated in a Microsoft Excel data sheet for analysis^30^. Identification codes were allocated to each individual dog. Dates of collection periods were recorded for each GPS dataset for each dog (some dogs were collared on more than one occasion in the NPA). Age (adult [>1 year old], puppy [≤1 year old], unknown), sex (male, female, unknown) and neuter status (neutered, entire, unknown) were standardised across the datasets. Region, community and the residence’s geographic coordinates were recorded for each dog. The coordinates of each dog’s residence were converted from geographic to projected coordinates (EPSG:28353: GDA94/MGA zone 53), and their locations confirmed by plotting them in geographic-information software (QGIS^31^). Dogs for which residence location was unknown were removed from the analysis.

GPS datasets for each dog were prepared for analysis. GPS fixes were removed prior to when the collar was fitted on the dog, after the collar was removed from the dog, and if the data was implausible for free-roaming activity (travel by vehicle indicated by speed >20 km/h, or GPS measurement error indicated by single outlier fix). GPS fix coordinates were projected (EPSG:28353: GDA94/MGA zone 53).

### Analysis

All analyses were implemented in the R statistical platform^32^ and analysed using the packages plyr^33^, ggplot^34^, multilevel^35^, lubridate^36^ and splancs^37^.

### Demographics

Summary statistics described the number of dogs in each community and region, and their age and sex. Chi-squared tests were used to determine if there were significant (P ≤ 0.05) differences between sex, neuter status and age.

### Temporal activity

Distance from residence was calculated as the difference between residence location and GPS fix location for all GPS fixes. Summary statistics described mean distance from residence for all dogs. Mean distance from residence stratified by community, region, month and year of collection, age, neuter status and sex were also summarised and visualised using boxplots.

### Daily pattern of activity away from dogs’ residences

The likelihood that dogs were away from their residence at different times of the day was investigated using von Mises kernel density plots (circular distributions of distance from home over a 24 hour period; R package overlap^38^). Data points >44 metres away from the residence address of the dog were categorised as ‘away from residence’, to account for GPS error^24^. Density plots were stratified by region, community, age, sex and neuter status. To construct density plots, distance was aggregated to the mean distance from residence during seven-minute consecutive intervals for all data-sets. This interval was selected based on twice the mean actual fix interval for GPS units that were set to record fixes at 1-minute intervals (note that the actual fix interval is greater than the set interval due to the time taken to acquire enough satellites for a GPS fix). This avoided over-representation of GPS points from datasets in which GPS fix intervals were short (15 s; TS dogs).

Linear regression analyses were then conducted to further explore the daily pattern of activity away from dogs’ residences for each region. The dependent variable – mean hourly distance from residence – was regressed on hour of the day for each region. Coefficient plots were used to visualise the change in mean distance roamed from residence for a 24-hour period in each region.

### Risk factors for activity away from dogs’ residences

Regression analyses were conducted to explore associations between potential risk factors and the distance from residence. Dog identification was included as a random effect to account for multiple GPS datasets for some dogs who were monitored repeatedly in different years or seasons. For each dependent variable, univariable analysis was conducted to investigate the effect of community, month, year, sex, neuter status and the interaction between sex and neuter status on mean distance from residence. Biologically plausible combinations (for example, month and season, were not included together) of variables that were found to be significantly associated with distance from residence in the univariable analyses were included in multivariable regression models. Final models were selected based on minimising Akaike’s Information Criterion with correction for small sample size (AICc); variables that were no longer significant were removed if the AICc of the subsequent model was lower than the initial model. For multivariable analyses, month was re-categorized to season (dry season – May to October; wet season – November to April)^39^.

## Results

During the study period, 235 dogs were collared. After exclusion of dogs without recorded residence locations, 213 GPS telemetry datasets were available for analysis (TS = 70 datasets, NPA = 143 datasets). The total number of individual dogs that were monitored was 132 (TS = 70 dogs, NPA = 62 dogs).

Table 2 shows the number of GPS datasets by age and sex for communities in each region. There was no significant difference in the proportion of dogs collared and datasets obtained in the two regions (X^2^ = 0.54, df = 1, P = 0.50). There was also no significant difference in the proportion of male and female dogs (X^2^ = 0.008, df = 1, P = 0.93), known neuter status (X^2^ = 0.63, df = 1, P = 0.43) or age (X^2^ = 2.57, df = 1, P = 0.11) between regions.

**Table 2:** Number of datasets by age and sex within regions and communities in a study of temporal roaming patterns of free-roaming domestic dogs using GPS-collars in the Torres Strait and Northern Peninsula Area, Queensland, Australia.

			Datasets (by age)		Datasets (by sex)	
Region	Community	Datasets (total)	Adult	Puppy	Male	Female
TS	Kubin	24	18	6	11	13
	Saibai	25	22	3	16	9
	Warraber	21	15	6	13	8
Region total		70	55	15	40	30
NPA	Bamaga	39	37	2	27	12
	Injinoo	16	16	0	1	15
	New Mapoon	30	25	5	17	13
	Seisia	32	32	0	10	22
	Umagico	26	25	1	11	15
Region total		143	135	8	66	77
Study total		213	190	23	106	107

**Figure 2:**
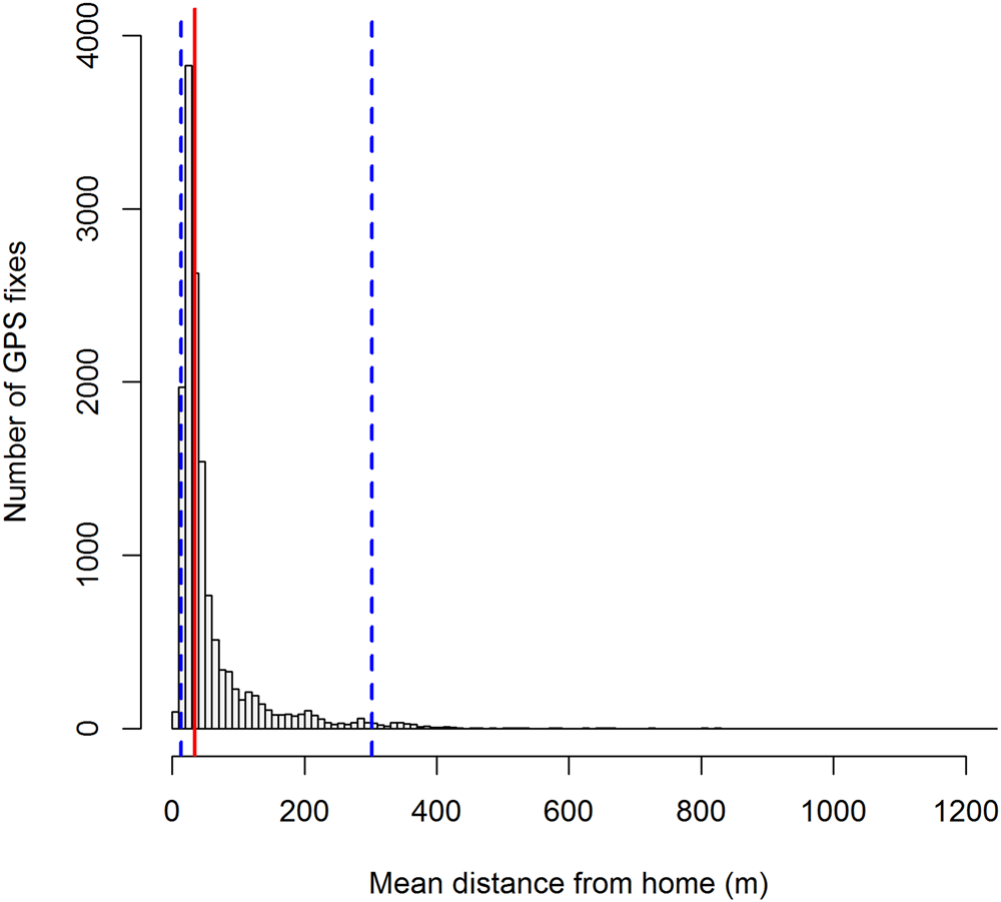
Histogram of mean distance from residence at which GPS fixes were recorded in a study of the temporal roaming patterns of free-roaming domestic dogs in the Torres Strait and Northern Peninsula Area, Queensland, Australia. The red line represents median distance and blue dashed lines represent 1^st^ and 3^rd^ quartiles (interquartile range).

**Figure 3:**
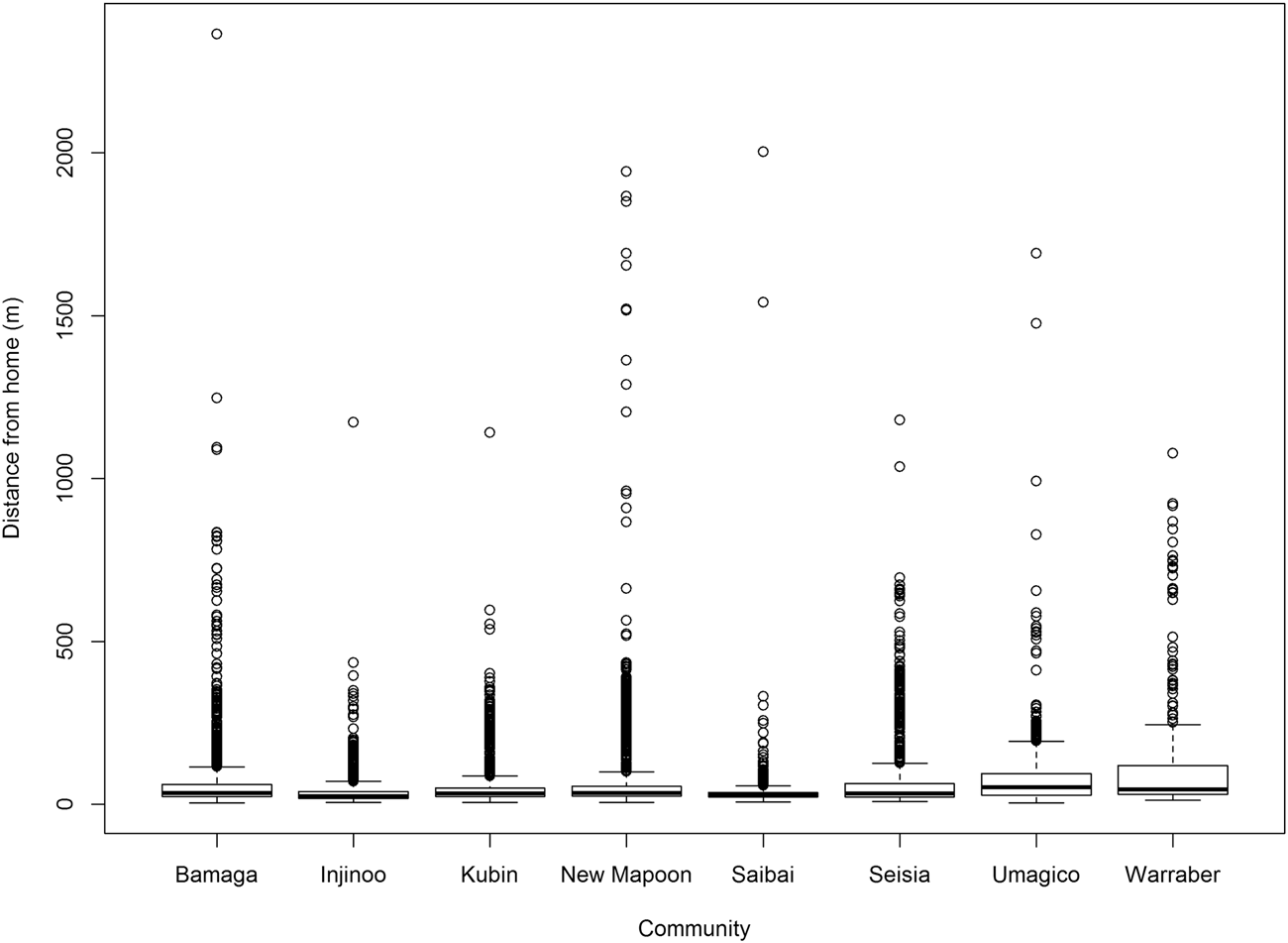
Boxplot of mean distance from residence recorded by community in a study of the temporal roaming patterns of free-roaming domestic dogs using GPS collars in the Torres Strait and Northern Peninsula Area, Queensland, Australia.

**Figure 4:**
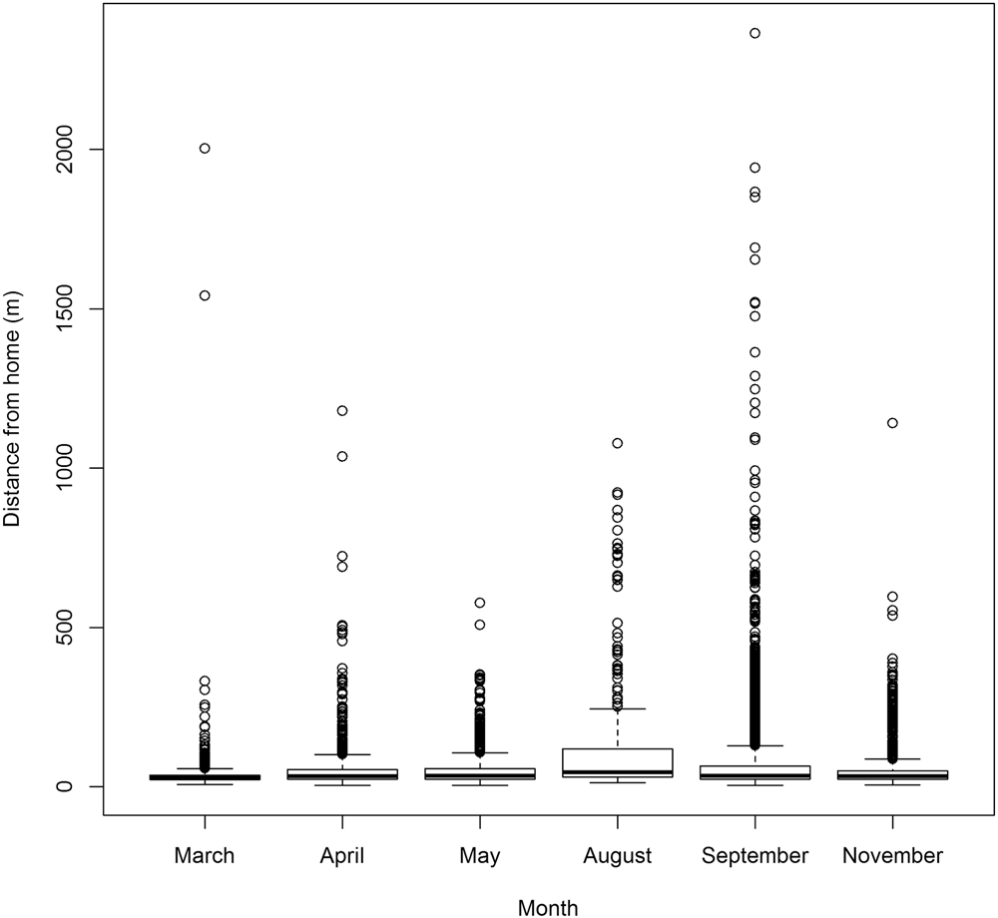
Boxplot of mean distance from residence recorded by month in a study of temporal roaming patterns of free-roaming domestic dogs using GPS collars in the Torres Strait and Northern Peninsula Area, Queensland, Australia.

The median distance from residence of all GPS fixes was 63 m (95% range 13–302 m; Fig. 2). Warraber had the largest interquartile range (IQR), whilst Saibai and Injinoo the smallest (Fig. 3). Umagico and Warraber dogs had the highest median distance from residence (54 m and 46 m, respectively), whilst Saibai and Injinoo had the lowest (28 m and 26 m, respectively; Fig. 3). Injinoo and Saibai had outliers furthest from the median (Fig. 3). By month, the lowest median distance from residence was in March (median 28 m) and greatest median distance was in August (median = 46 m; Fig. 4).

### Daily pattern of activity away from dogs’ residences

**Figure 5:**
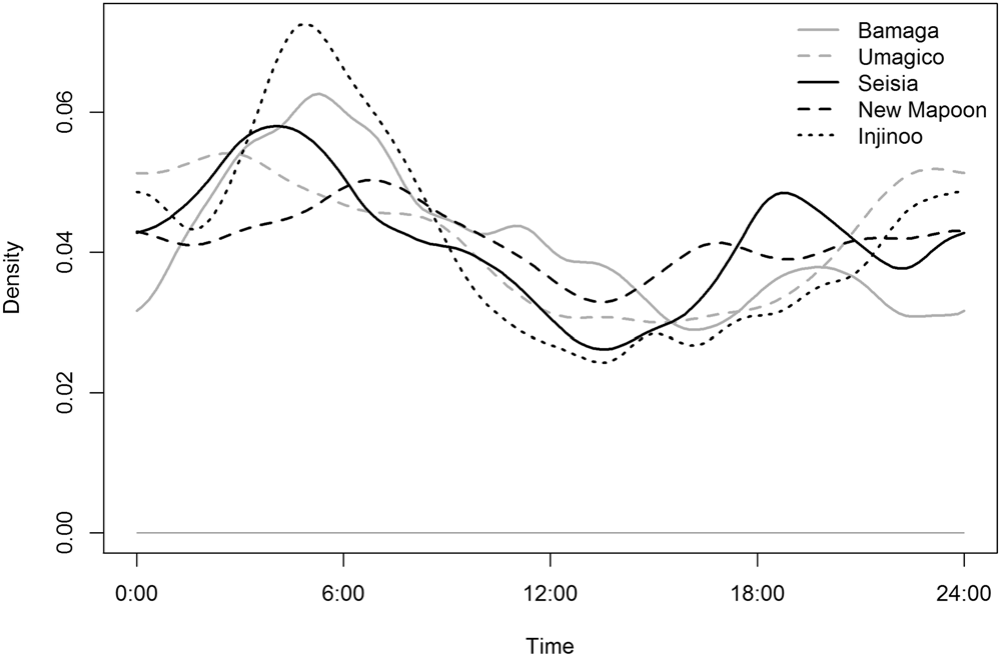
Density plot of the proportion of dogs away from their residence (>44 m) in a study of temporal roaming patterns of free-roaming domestic dogs using GPS collars in the Northern Peninsula Area, Queensland, Australia.

**Figure 6:**
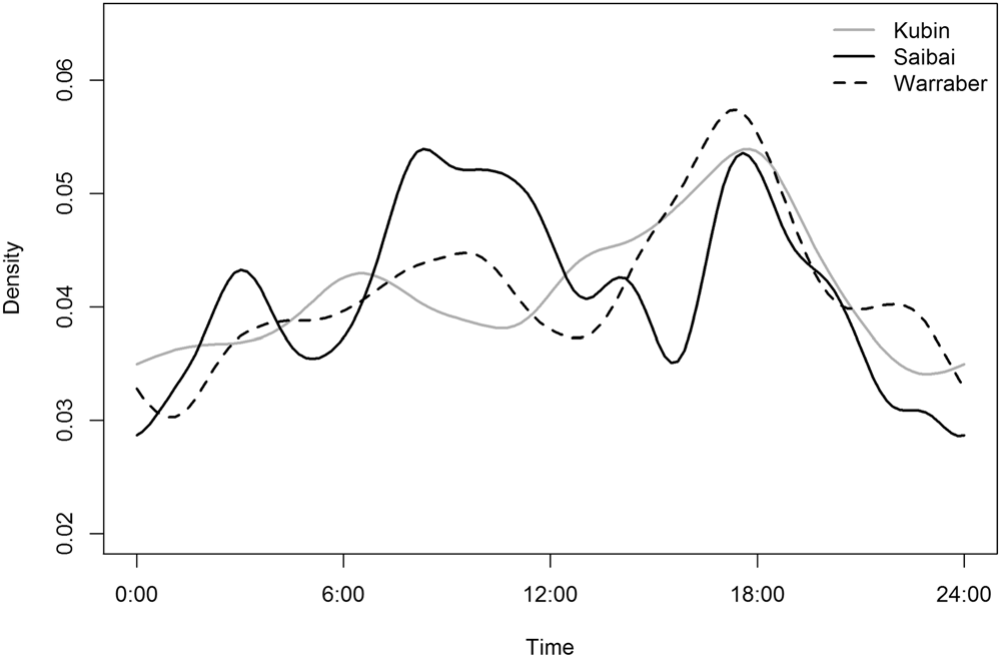
Density plot of the proportion of dogs away from their residence (>44 m) in a study of temporal roaming patterns of free-roaming domestic dogs using GPS collars in the Torres Strait, Queensland, Australia.

Density plots of the proportion of dogs away from their residence (>44 m) are shown in Figs 5 and 6. There was a general pattern of two peaks when dogs were away from their residences during the 24-hour period in all communities in the NPA at approximately 0600 hrs and 1800 hrs; the largest morning peak was in Injinoo and the largest evening peak was in Seisia (Fig. 5). The lowest morning peak was in New Mapoon and the lowest evening peak was in Bamaga (Fig. 5). The highest proportion of dogs were at their residence at around 1400 hrs in all communities except Bamaga (approximately 1700 hrs).

In the TS, the proportion of dogs away from their residence increased from midnight to reach an evening peak at 1800 hrs (Fig. 6); the highest evening peak was observed in Warraber. Dogs were most likely to be at their residence at midnight.

Both male and female dogs displayed peaks away from their residence at 0600 hrs and 1800 hrs (Supplementary Information, Fig. S1). Neutered and entire dogs, as well as those of unknown neuter status, also displayed the same peaks (Supplementary Information, Fig. S2), as did both adult dogs and puppies (Supplementary Information, Fig. S3).

**Figure 7:**
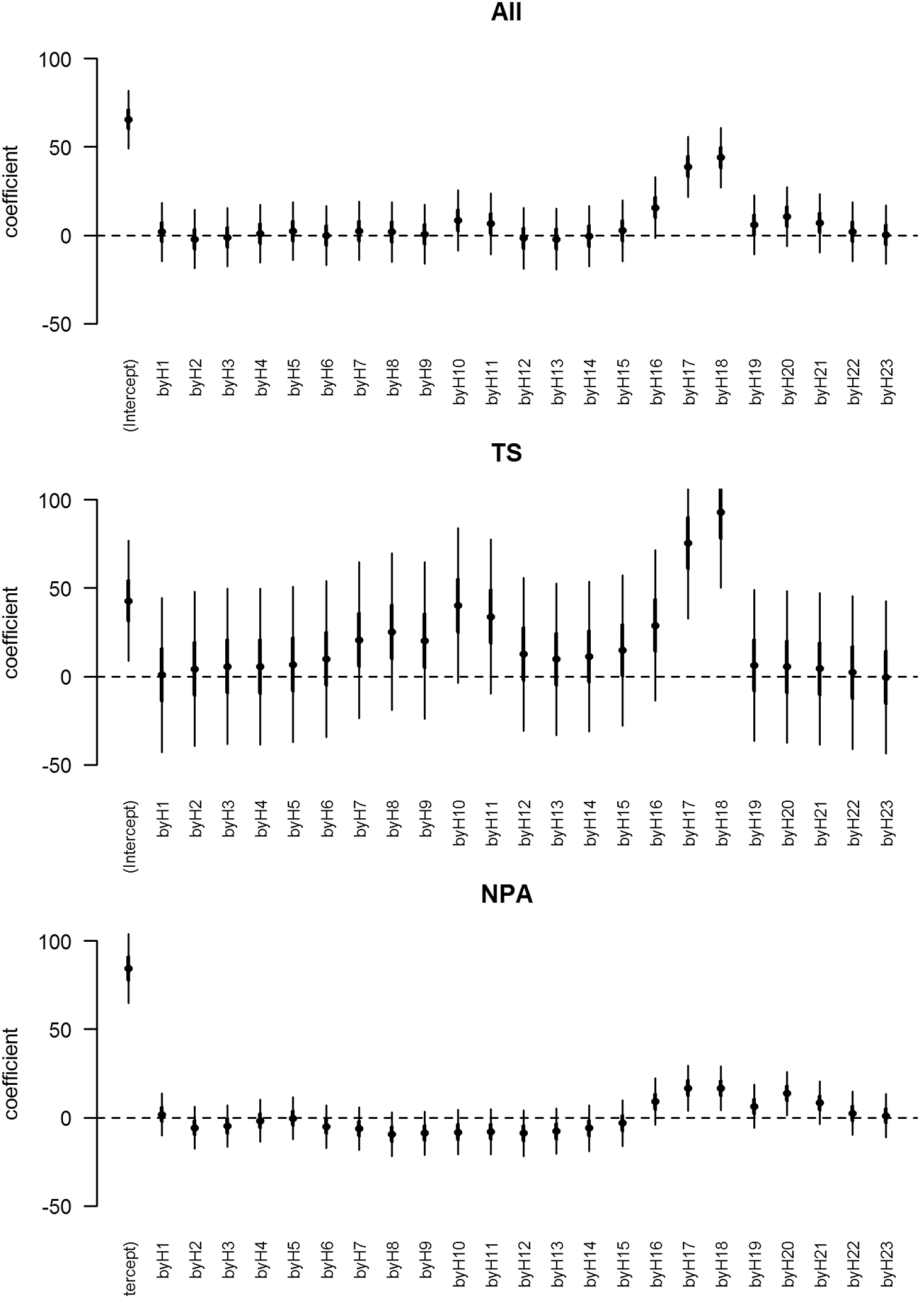
Coefficient plot of distance from residence by hour in a study of the temporal roaming patterns of free-roaming domestic dogs in the Torres Strait, Queensland, Australia. The coefficient is measured in meters. NPA = Northern Peninsula Area, TS = Torres Strait.

Regression analysis showed that hour of the day was significantly associated with the mean distance from residence (P < 0.001) in each region. Consistent with the density plots, there were two main peaks of mean distance from residence in the TS, with one morning peak around 1000 hrs to 1100 hrs, and one evening peak around 1700 hrs to 1800 hrs. Mean distances from residence were 83 m and 77 m at 1000 hrs and 1100 hrs in the morning, respectively, and 118 m and 136 m at 1700 hrs and 1800 hrs in the evening, respectively (Fig. 7). In the NPA there was only one main peak in the evening, from 1700 hrs to 1800 hrs, with mean distances from residence of 101 m at both 1700 hrs and 1800 hrs (Fig. 7).

### Risk factors for activity away from dogs’ residences

Univariable regression analysis demonstrated that community, month and year were significantly associated with distance from residence (Table 3). The mean distance from residence recorded for Saibai dogs was 34 m. Dogs in Injinoo, Kubin and Seisia roamed a similar distance, whilst dogs in Warraber, New Mapoon, Bamaga and Umagico roamed significantly further. The dogs that roamed the furthest mean distance from their residence were from Umagico (102 m). Dogs roamed furthest in May and September and least in March and November; they also roamed furthest in 2013 and 2014 and least in 2016 and 2017. Sex, neuter status and age were not significantly associated with distance from residence. Given the timing of studies (NPA communities were generally studied in 2013 and 2014, and TS communities in 2016 and 2017), we expected that the effect of year was due to the effect of community. Therefore, ‘year’ was not included in the multivariable regression analyses.

**Table 3:** Univariable regression analysis summary of fixed effects of variables associated with mean distance from residence in a study of the temporal roaming patterns of free-roaming domestic dogs in the Torres Strait and Northern Peninsula Area, Queensland, Australia.

Variable	Level	Number in level	Coefficient (m)	Standard error (m)	P value	AICc
Community					<0.01	167801
	Saibai^†^	25	34.4	12.0		
	Injinoo	16	16.6	26.2	0.53	
	Kubin	24	23.0	16.9	0.17	
	New Mapoon	30	30.1	19.6	0.13	
	Warraber	21	43.8	17.6	0.01	
	Seisia	32	59.7	21.1	0.01	
	Bamaga	39	55.0	18.8	<0.01	
	Umagico	26	67.6	19.2	<0.01	
Sex					0.21	—
	Female^†^	107	61.8	8.0		
	Male	106	13.4	10.6	0.21	
Neuter status					0.14	—
	Entire^†^	136	64.2	6.3		
	Neutered	62	8.5	12.6	0.50	
	Unknown	15	38.6	19.5	0.05	
Age					0.32	—
	Puppy^†^	23	57.3	13.1		
	Adult	190	14.3	14.4	0.32	
Month		<0.01	167818			
	March^†^	25	34.4	12.0		
	Nov	24	23.0	16.9	0.17	
	April	32	43.7	14.3	<0.01	
	Aug	21	43.8	17.6	0.01	
	May	30	44.9	14.3	<0.01	
	Sep	81	51.0	14.1	<0.01	
Year					<0.01	167831
	2017^†^	25	34.4	12.2		
	2016	45	32.5	15.0	<0.01	
	2013	46	45.7	14.4	0.03	
	2014	97	51.7	14.3	<0.01	

AICc = Akaike’s Information Criterion (corrected for small sample size). ^†^Reference level.

Because both region and community were significantly associated with distance from residence in the univariable models, two models were investigated using multivariable regression analysis and the following combinations of risk factors: 1. *community* and *season* (month reduced to season [Wet or Dry] to enable convergence of models), and 2. *region* (NPA or TS) and *season*. Both community and season in Model 1 were significantly associated with distance from residence, and this model had the lowest AICc of all models, including univariable models, in which variables were significantly associated with distance from home (Table 4). Roaming distances by community were similar to those in the univariable analysis. Dogs roamed a mean of 5.5 m further in the dry season. Region and season were also both significantly associated with distance from residence in Model 2 and again, this model had lower AICc than univariable models (Table 5). Overall, dogs in the NPA were estimated to roam further than those in the TS region. Again, dogs were estimated to roam further (mean 6.3 m) in the dry season in both regions.

**Table 4:** Summary of fixed effects of multivariable regression analysis of variables associated with mean distance from residence in a study of the temporal activity of free-roaming domestic dogs using GPS collars in the Torres Strait and Northern Peninsula Area, Queensland, Australia.

Variable	Level	Coefficient (m)	Standard error (m)	P value	AICc
Intercept	Saibai, wet season^†^	34.4	12.0	<0.01	167796
Community				0.01	
	Injinoo	11.7	26.3	0.66	
	Kubin	23.0	16.8	0.17	
	New Mapoon	25.3	19.8	0.20	
	Warraber	38.3	17.8	0.03	
	Bamaga	50.2	19.0	0.01	
	Seisia	55.2	21.2	0.01	
	Umagico	62.4	19.4	<0.01	
Season				0.05	
	Dry	5.5	2.8	0.05	

AICc = Akaike’s Information Criterion (corrected for small sample size). ^†^Reference level.

**Table 5:** Multivariable regression analysis summary of fixed effects of variables associated with mean distance from residence in a study of the temporal roaming patterns of free-roaming domestic dogs in the Torres Strait and Northern Peninsula Area, Queensland, Australia.

Variable	Level	Coefficient (m)	Standard error (m)	P value	AICc
Intercept	TS, Wet season^†^	53.9	7.2	<0.01	167841
Region				0.01	
	NPA	24.4	10.4	0.02	
Season				0.02	
	Dry	6.3	2.8	0.02	

AICc = Akaike’s Information Criterion (corrected for small sample size). ^†^Reference level.

## Discussion

This study indicates that free-roaming domestic dogs’ use of their environment in communities in the Torres Strait (TS) and Northern Peninsula Area (NPA) of Queensland, Australia varies by time of the day, season and location. There are many potential drivers of roaming from residence by dogs in communities in which free-roaming is the norm. For example, roaming might be associated with human activity. In a study of urban free-roaming owned dogs in Chile, GPS fixes were most frequent between 12:00 and 16:00 when people returned home for lunch^40^. It was hypothesised that at other times, dogs were more likely to be asleep under cars or trees that obstructed sky-view for GPS fixes. Dogs in both the TS and NPA roamed furthest in the evening when people are likely to have returned from work or school and travel around the community for activities such as shopping, visiting friends and relatives, and engaging in recreational activities. The dogs might be inclined to follow their owners around the community, resulting in a greater proportion of dogs roaming larger distances at these times. Dogs also did not roam as far in the morning and middle of the day as they did in the evening. This could also be associated with human activity – which is likely to be reduced at these times – but might also be due to the higher temperatures in the middle of the day, causing dogs to seek shade.

Besides human activity, dogs might also roam to engage in dog-orientated activities such as seeking mates and food, and patrolling territory. We did not find that sex or neuter status influenced the distance that dogs roamed away from their residence, which might have been expected if mate-seeking behaviour influenced roaming distance. However, due to the cyclicity of reproductive activity in dogs (generally a 6 to 9-month reproductive cycle in females), it is possible that the number of datasets were too few and of insufficient duration to detect sex-specific differences in roaming distance from residence. Previous analysis of more geographically diverse GPS datasets from dogs in northern Australia found that entire males generally had the largest home-ranges, a finding which supports this hypothesis but could also be caused by entire males patrolling their territory^22,25^. In a study of 14 livestock guardian dogs (LGD) on three large farms in Victoria, Australia, it was found that the LGDs had morning and afternoon peaks of activity^41^. The sheep that the dogs guarded also had morning and afternoon activity peaks, but unlike the sheep, the dogs were also more active at night than during the day. Overall, the dogs’ activity pattern was more consistent with the activity times of the main predator species. Therefore, the authors hypothesised that the dogs’ activity was most likely associated with territory patrol and protection of their sheep, rather than directly associated with the activity of the sheep. In the current study, territory patrol might also drive the peaks of activity of the dogs; wild dogs are present in the NPA and domestic dogs might perceive them as a threat in the early morning, whilst in both the Torres Strait and the NPA, human activity might be more influential on the distance that dogs roam in the evenings.

The potential overlap of domestic and wild dog home ranges and roaming activity is important in the context of rabies preparedness. In communities in which domestic dogs roam large distances – such as those in the NPA – there is a risk of disease spread to susceptible wildlife and feral species^12^. This highlights the need for effective movement controls in NPA communities, particularly in the early evening when there is both a greater proportion of dogs that are away from their residence, and those dogs are at greater distances from their residence. Some species such as feral pigs or cats are spill-over hosts for rabies, and whilst it is possible that infected individuals could present some risk (for example to hunters and their dogs^42^), these populations are unlikely to become a reservoir of canine-rabies and present ongoing risks. However, wildlife canid populations such as red foxes are important hosts of rabies globally, and eradication of rabies in these populations is challenging^43,44^. Although there are no foxes in the study region, it is possible that the wild dog population could become a reservoir for rabies following spread from domestic dogs (or following a direct incursion into the wild dog population) in regions such as the NPA^17,45^. Such a situation is likely to be difficult to resolve and result in the need for ongoing vaccination of domestic dogs – requiring vigilance to maintain – as well as post-exposure prophylaxis of people following bites, and potential livestock losses.

Differences in dogs’ daily activity periods between regions and communities is also consistent with findings from other studies of free-roaming dogs, both in Australia and worldwide^27,40,46^. For example, Rubin and Beck (1982) found that free-roaming dogs in areas of Queens, New York, were most active in the early morning, with lower but more consistent activity later in the day. In a study in the Tiwi Islands by Kennedy *et al*.^27^, peak roaming occurred only in the morning, rather than in both the morning and evening. As well as differences in the need for territory patrol as described above, different human activity or food availability between regions and communities might also exist. In another study, owned free-roaming dogs have been shown to have smaller home ranges than stray dogs, which was suggested to be caused by owners’ influence on their behaviour and subsequent territory size^47^. In addition, environmental differences might also influence regional and community variation in roaming distances. Dogs in the NPA were found to roam furthest, perhaps because of more available land in the NPA compared to the smaller TS island communities. In particular, Saibai community is surrounded by swamp, mangrove and ocean without reef (unlike Warraber, where dogs roam on the reef)^28^ which is likely to restrict the distance roamed. Regardless of the reasons, community and regional differences in roaming distances and time of day indicates the need for response strategies tailored to individual communities or regions to ensure sufficient management of dog interactions to reduce disease transmission in the case of a rabies incursion.

Dogs were also found to roam further in the dry season in both the community and region models in the current study. Previous research has also indicated that season influences dog activity. In a study of LGDs in Victoria, Australia^41^, dogs’ peaks of activity were at sunrise and sunset and therefore, the times of day shifted with season due to the southerly latitude of the study location. In northern Australia, the nature of the wet season – monsoonal rains and areas of standing water – might present barriers to roaming, and could have restricted dogs’ distance from their residence in the current study, Consistent with this, in a study of the home range size of free-roaming domestic dogs in more diverse locations in northern Australia than the current study, home ranges were larger before the wet season than after^25^. We expect that spread of a disease such as rabies might be facilitated in the dry season in northern Australia, because greater roaming distances could increase the opportunity for interactions between dogs, as well as contact with susceptible wildlife. It is therefore important to consider the need for an increased focus on movement controls following an incursion in the dry season.

There are some limitations of the current study. Selection bias due to the opportunistic selection of dogs from owners who were present in the community and willing to participate might have influenced findings. Dogs with a lower propensity to roam the community might have been selected because they were more likely to be at their residence, resulting in under-estimation of roaming distances in these dog populations. Possible risk factors not considered in this study include differences in human activity, food availability, breeding season and dog breed. These factors might have influenced roaming distance or times and should be considered in further investigations. For example, the collection of data throughout a range of months might provide more insight about the influence of mate-seeking behaviour. In the TS, fewer datasets were included in the study, which might have reduced precision of estimations and power to detect significant differences between risk factors. GPS error might also have affected the precision of estimates, but it is unlikely that GPS error was greater in one of the regions compared to the other.

In conclusion, we identified that location, season and hour of day were significantly associated with the roaming activities of free-roaming domestic dogs. When planning rabies control strategies – such as dog movement controls or the availability of dogs for vaccination – these factors should be taken into consideration. In the regions in this study, movement controls should be targeted to the early evening, when the greatest proportion of dogs are roaming at the furthest distances from their residences. Increased rigor of movement controls of domestic dogs and surveillance of wildlife is also required in the dry season, when dogs roam further.

## Supplementary information

Supplementary Information
