# Supplementary material for: Investigation of the temporal roaming behaviour of free-roaming domestic dogs in Indigenous communities in northern Australia to inform rabies incursion preparedness
Source: Sci Rep. 2019 Oct 17;9:14893. doi: 10.1038/s41598-019-51447-8 (PMC6797733; doi:10.1038/s41598-019-51447-8)
Supplement: Supplementary file 1 — Supplementary Information [file 41598_2019_51447_MOESM1_ESM.pdf]

Investigation of the temporal roaming behaviour of free-roaming domestic dogs in Indigenous communities in northern Australia to inform rabies incursion preparedness

Elizabeth K. Maher[1], Michael P. Ward[1*], Victoria J. Brookes[1,2]

1 Sydney School of Veterinary Science, The University of Sydney, Camden, Australia

2 School of Animal and Veterinary Sciences, Charles Sturt University, Wagga Wagga, Australia

SUPPORTING INFORMATION

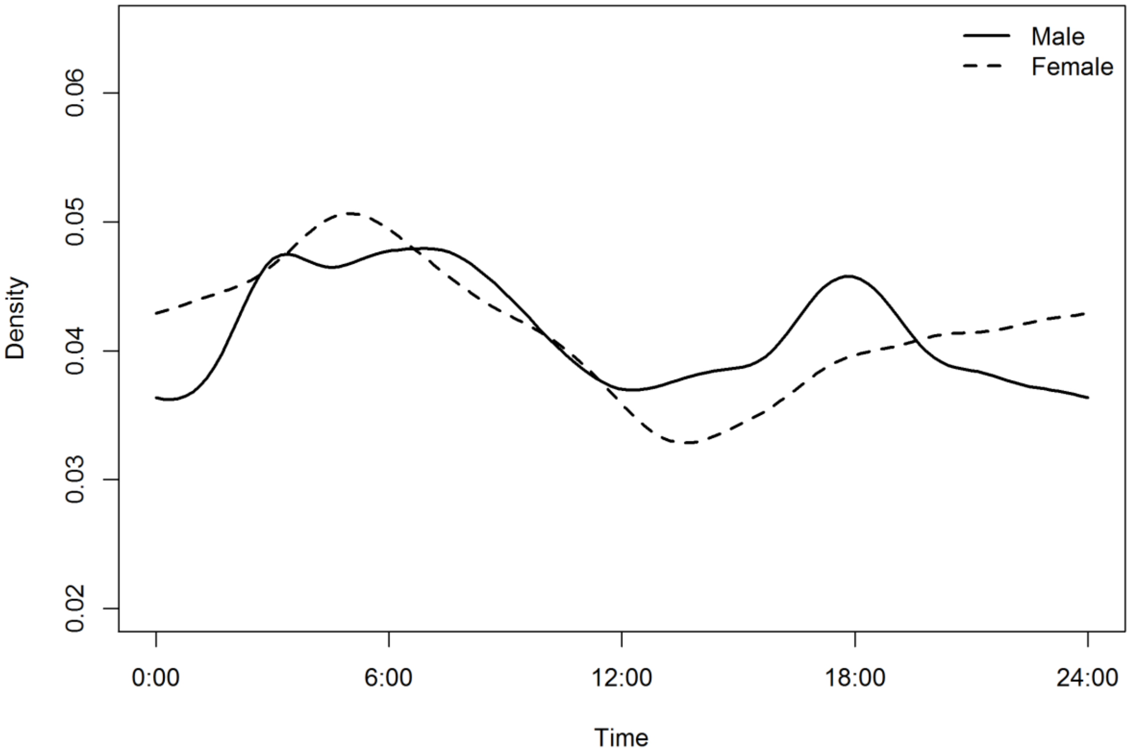

Figure S1: Density plot of mean distance from residence for dogs by sex in a study of the temporal activity of free-roaming domestic dogs using GPS collars in the Torres Strait, Queensland, Australia.

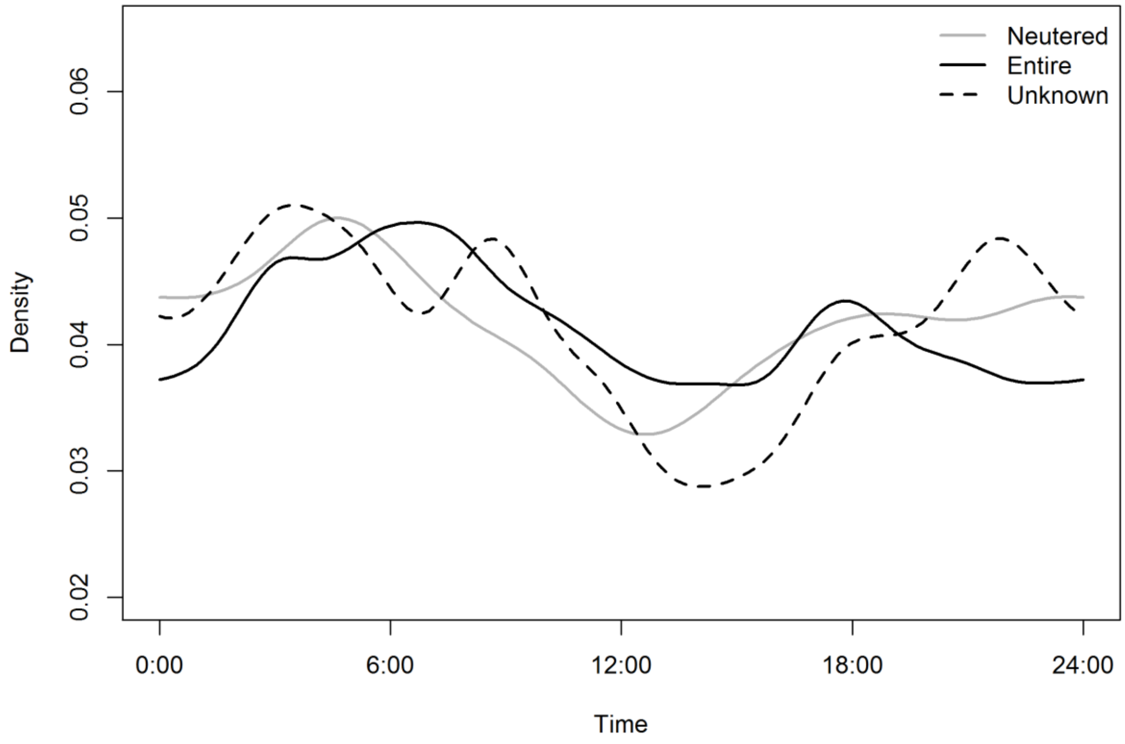

Figure S2: Density plot of mean distance from residence for dogs by neuter status in a study of the temporal activity of free-roaming domestic dogs using GPS collars in the Torres Strait, Queensland, Australia.

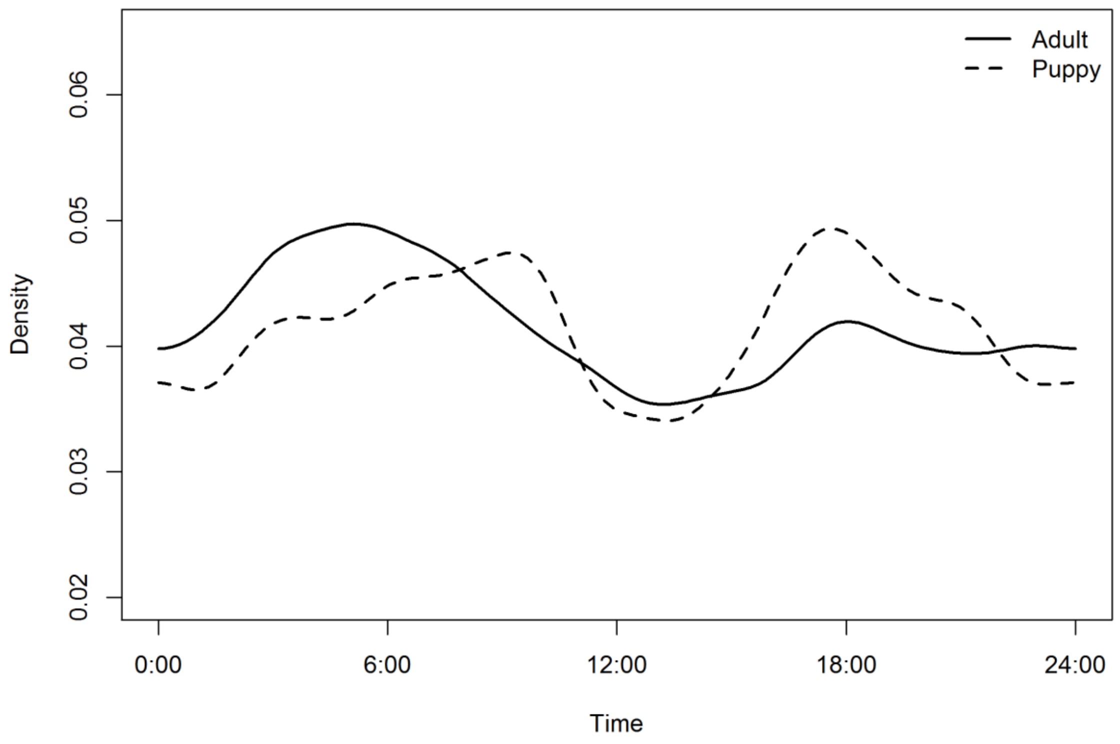

Figure S3: Density plot of mean distance from residence for dogs by age in a study of the temporal activity of free-roaming domestic dogs using GPS collars in the Torres Strait, Queensland, Australia.
